# Determining similarity in histological images using graph-theoretic description and matching methods for content-based image retrieval in medical diagnostics

**DOI:** 10.1186/1746-1596-7-134

**Published:** 2012-10-04

**Authors:** Harshita Sharma, Alexander Alekseychuk, Peter Leskovsky, Olaf Hellwich, RS Anand, Norman Zerbe, Peter Hufnagl

**Affiliations:** 1, Electrical Engineering Department, IIT Roorkee, India; 2 Computer Vision and Remote Sensing Group, Technical University, Berlin, Germany; 3 Dept. Digital Pathology and IT, Institute of Pathology, Charité - Universitätsmedizin Berlin, Berlin, Germany

**Keywords:** Attributed Relational Graphs (ARG), Region of Interest (ROI), Breast tissue biopsy, Connected components, Graph-theoretic, A* search

## Abstract

**Background:**

Computer-based analysis of digitalized histological images has been gaining increasing attention, due to their extensive use in research and routine practice. The article aims to contribute towards the description and retrieval of histological images by employing a structural method using graphs. Due to their expressive ability, graphs are considered as a powerful and versatile representation formalism and have obtained a growing consideration especially by the image processing and computer vision community.

**Methods:**

The article describes a novel method for determining similarity between histological images through graph-theoretic description and matching, for the purpose of content-based retrieval. A higher order (region-based) graph-based representation of breast biopsy images has been attained and a tree-search based inexact graph matching technique has been employed that facilitates the automatic retrieval of images structurally similar to a given image from large databases.

**Results:**

The results obtained and evaluation performed demonstrate the effectiveness and superiority of graph-based image retrieval over a common histogram-based technique. The employed graph matching complexity has been reduced compared to the state-of-the-art optimal inexact matching methods by applying a pre-requisite criterion for matching of nodes and a sophisticated design of the estimation function, especially the prognosis function.

**Conclusion:**

The proposed method is suitable for the retrieval of similar histological images, as suggested by the experimental and evaluation results obtained in the study. It is intended for the use in Content Based Image Retrieval (CBIR)-requiring applications in the areas of medical diagnostics and research, and can also be generalized for retrieval of different types of complex images.

**Virtual Slides:**

The virtual slide(s) for this article can be found here: http://www.diagnosticpathology.diagnomx.eu/vs/1224798882787923.

## Background

Histology may greatly benefit from development of suitable automatic analysis methods. Histological image analysis can contribute towards diagnosis and treatment planning, study and research work. Sometimes, it is required to find the similarity between histological images or their regions. Given a database of reference images and a query image, one or several images from the database need to be retrieved which are similar to the query. Content-based image retrieval (CBIR) can can address this problem, particularly using graph-based approach.

Pathologists make use of staining intensity, morphological changes and notably spatial relationships of tissue components during histopathological examinations. Designing a system which retrieves sample regions being structurally similar to a region in question can contribute towards automated detection of malignant changes. Besides research and education, clinical pathology is expected to benefit from such a system where visually interesting regions containing similar tissue structures can be selected and retrieved from existing large databases for further studies. Therefore, the work has been performed keeping in mind the generic nature of medical images as well as the specific nature of the histological data to be analysed, by exploiting the representational power of graphs to describe such complex images efficiently.

Tagare et al. have presented a content-based retrieval approach for medical image database in [1], where it has been strongly emphasised that medical image information contains spatial data and a large part of image information is geometric. The state-of-the-art general-purpose CBIR techniques using low-level features based on texture, colours and shape are insufficient for histological images since these methods do not incorporate high-level structural information and neighbourhood relationships between image regions. Therefore, an appropriate improvement in this direction can be the use of structural methods adopting graphs, being explored in this paper.

Graphs have recently drawn increasing attention of the scientific community as effective structural descriptors due to their ability to represent relational information. They can be employed for providing efficient descriptions of images by associating nodes with specified attributes to image components and edges with appropriate weights to relationships between these components. This property can be exploited to obtain graph-based representations of the database and the query images, and then to search for structurally similar images by means of inexact graph matching, which involves calculation of a matching cost. The closest matches can then be obtained and displayed in decreasing order of similarity (i.e. increasing cost of matching). Hence, the aim of this work is to provide an algorithm for automatic content-based retrieval of similar images from large histological databases, which, at such scale, would not be feasible to perform only by visual analysis of humans.

In order to analyse histological images for diagnostic purpose, a semi-automatic method using low-level features of tissue images has been proposed in [2] for automatic selection of ROIs for further diagnosis. Kayser et al. [3] discuss the information recognition algorithms that can be used for field of view detection in virtual microscopy, by measuring diagnosis-relevant information. They include graph representations of tissues based on Voronoi diagrams. Some classification methods have been developed as tools for diagnostic assistance in histopathological examinations of lungs [4,5].

Graph theory has also been used by authors for information representation in the field of histology. The most common method is Delaunay triangulations (and their corresponding Voronoi diagrams) where nuclear components of the tissue are considered as graph nodes [6,7]. Minimum spanning trees can also be obtained from them. Probabilistic graphs where nucleus forms nodes and edges are assigned according to some probability distribution have been proposed in [8]. However, all these graphs exhibit low-level (pixel-based) information of the image, unlike graphs introduced in this work as they contain high-level (region-based) information related to structure and spatial relationships between regions.

### Overview of Content Based Image Retrieval

In Information Retrieval (IR) systems, the user specifies a query either in the form of text, documents, images, or sounds and the system is expected to return the items that are semantically similar to the query in some sense. CBIR is an information retrieval system that includes techniques for retrieving digital images by their visual content. The horizon of CBIR includes methods ranging from image similarity functions to highly complex image annotation systems [9].

At present, CBIR is an extremely active area of research. Descriptions of a variety of CBIR approaches implemented in the past are given in reviews [10] and [11]. CBIR has been applied to medical domain and a comprehensive review on medical CBIR systems is given by Müller et al. [12]. However, most of the recently developed retrieval methods are dedicated to radiological images [13]. Specifically for histological images, research in this field has been comparatively less. An application with histopathological images is described in [14], using a property concept frame representation for morphological characteristics based on fuzzy logic. However, it does not emphasize on the spatial relationships between the various tissue components, which are considered an important aspect in our work, in order to describe the overall topology of the breast tissues.

In Diamond Project [15], the interactive search in large distributed data repositories was addressed. Particularly, the most relevant to medical domain are MassFind [16], FatFind [17] and PathFind [18]. MassFind is an application for diagnosing lesions in mammograms, which focuses on performance of different distance metrics to define similarity between ROI images. FatFind exploits the property of perfect round shape of adipocytes in cell microscopy images for their automatic counting, by making use of low-level shape features of cells. PathFind is a tool employing “discard-based search” for content-based retrieval of WSIs. However, in all the applications, less attention is given to high-level structural representation and retrieval algorithms specific to histological images; but more emphasis is on development of design and implementation strategies of the search methods, to handle huge data collections in large-scale efficient network-distributed frameworks.

The images used in CBIR systems for a particular application form a **Domain-Specific Collection**. It is the term given to a homogeneous collection of images *“providing access to controlled users with very specific objectives.”*[9]. For instance, satellite and biomedical image databases form two such collections. Histological images of breast tissues is also a domain-specific collection. The two main steps in CBIR include: 

1. **Signature calculation:** Mathematically describing images based on the characteristics of their visual content. The mathematical description is called “signature” and may include intensity, colour, texture, shape, size, location or their mixtures [19]. The signature must be selected carefully, depending on the context, as it describes the content within the image.

2. **Similarity measure calculation:** Assessing the similarity between a pair of images (query and database) and retrieving those database images having highest similarity to the query submitted to the system.

In the proposed method, signatures of the histological images are attributes of nodes and edges obtained from the graph-theoretic representation of the images as well as the topology of the graph. The corresponding similarity calculation is achieved by graph matching method and the obtained matching costs employed for retrieving images structurally close to the query image from the database. There are two types of tasks included in CBIR: off-line and on-line [20]: 

1. The **off-line** task includes feature extraction from and signature calculation of the database images, as well as the storage of the computed signatures. At this stage, there is no interaction with the user for retrieval task.

2. The **on-line** task includes analysis of the query image and its signature calculation. It also includes similarity computation, search and retrieval of similar database entries as well as interaction with the user through a GUI.

### Graph Theory

A graph is a set containing a finite number of points, called nodes (or vertices), which are connected by lines called edges (or arcs). In this paper, a graph is considered as a 4-tuple *G*=(*V*,*E*,*α*,*β*), where 

• *V* is the finite set of vertices.

• *E *⊆ *V *× *V* is the set of edges.

• *α*:*V *→ *L* is a function assigning labels to the vertices.

• *β*:*E *→ *L* is a function assigning labels to the edges.

**Figure 1:**
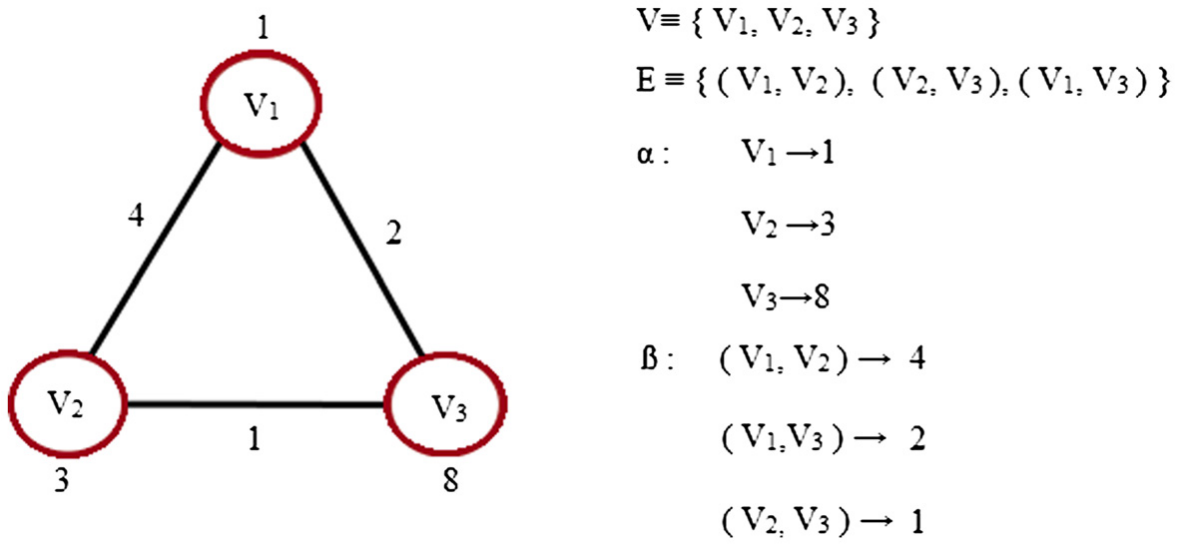
**A graph example.** An example of a basic graph represented as a 4-tuple *G*(*V*,*E*,*α*,*β*), with functions *α *and *β* defining the labels of nodes and edges, respectively.

Figure 1 gives an example of a basic graph.

#### Attributed Relational Graph

**Figure 2:**
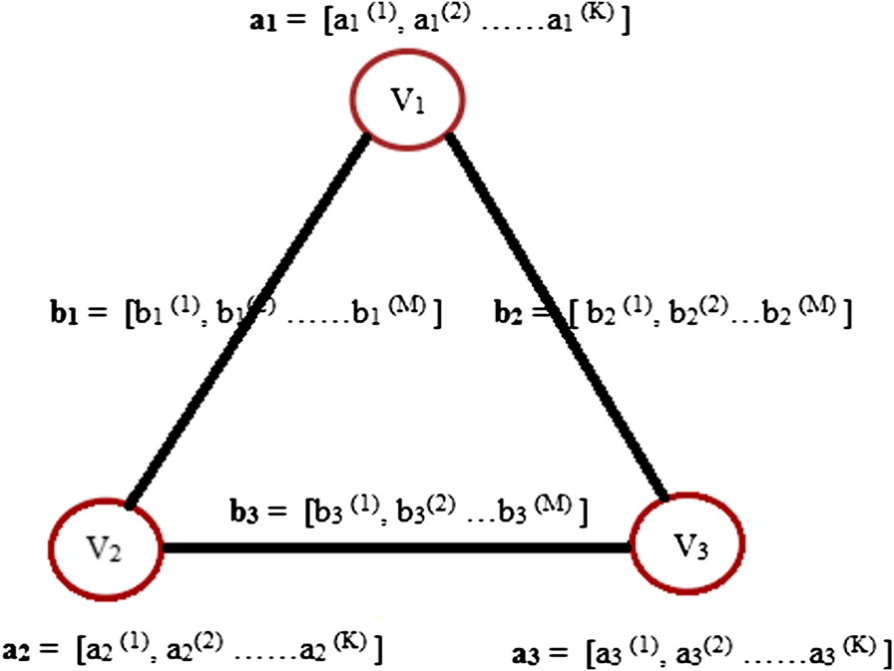
**Attributed Relational Graph.** An example of an ARG with node attribute vectors *a*_*i*_,*i*=1,2,3 and edge attribute vectors *b*_*j*_,*j*=1,2,3.

A graph *G* is said to be an Attributed Relational Graph (ARG) when both the nodes and the edges are represented with attributes. The node attributes for node *n*_*i*_ are denoted as a vector $a_{i}=[a_{i}^{\left(k\right)}],(k=1,2,3,\ldots ,K)$, where *K* is the number of node attributes in the vector **a**_**i**_, and the edge attributes (or weights) for edge *e*_*j*_ by the vector denoted as $b_{j}=[b_{j}^{\left(m\right)}],(m=1,2,3,\ldots ,M)$, where *M* is the number of edge attributes in the vector **b**_**j**_. In Figure 2, ARG with node attribute vectors **a**_**i**_, *i*=1,2,3 and edge attribute vectors **b**_**j**, _*j*=1,2,3 is shown. Node attributes represent quantities such as size, position, shape and colour of an object whereas edge attributes define relationships between nodes like the distance between two points or dissimilarity between objects. ARGs act as convenient structures for physical representation and are frequently used in applications ranging from computer-aided design to machine vision [21]. ARGs have been employed in this work for representing the information content of histological images.

#### Regional Adjacency Graph

**Figure 3:**
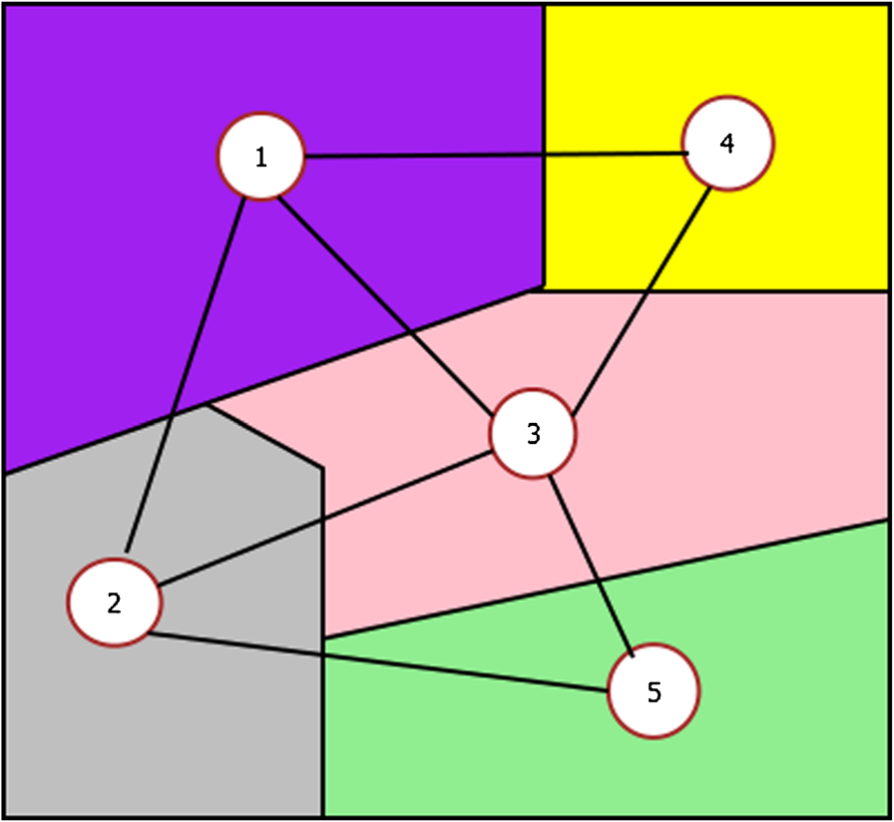
**Regional Adjacency Graph example.** An RAG is constructed over a simple image consisting of five regions.

Regional Adjacency Graph (RAG) is an ARG whose vertices represent regions and edges represent connections between adjacent regions. Node attributes are assigned according to characteristics of the region corresponding to each node and edge attributes (or weights) describe the adjacency relationships. RAGs give a spatial view of the images and are effective in applications for representing image information where neighbourhood relationships can be taken into account. RAGs have been used in [22] for segmentation of colour images. In this work, segmented histological images are represented as RAGs. Figure 3 shows a simple RAG example.

### Graph Matching

**Figure 4:**
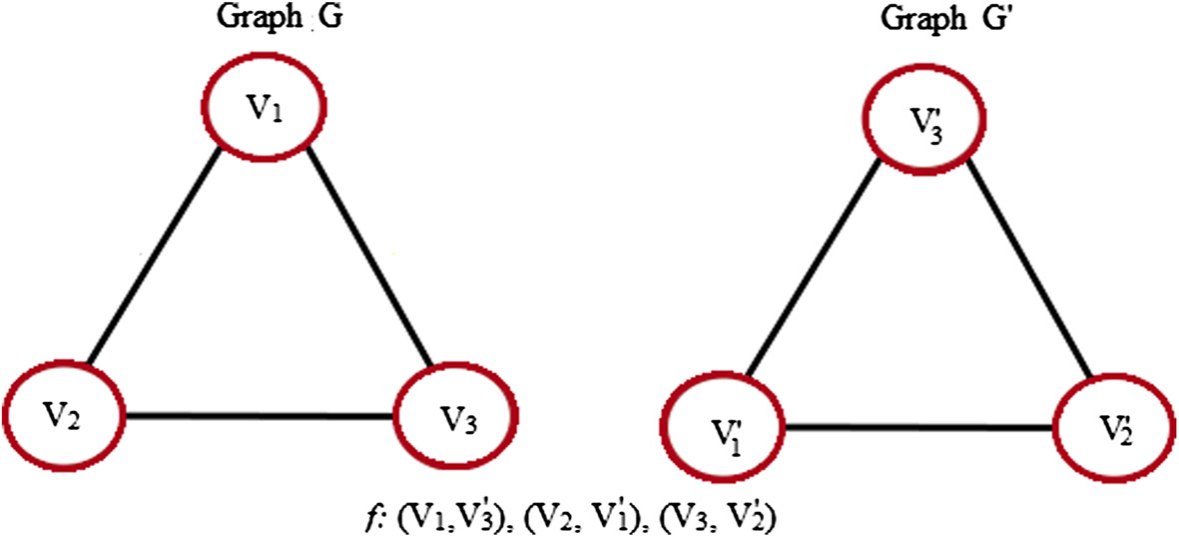
**Graph isomorphism.** An example of two isomorphic graphs G and G’, each having three nodes and three edges.

**Figure 5:**
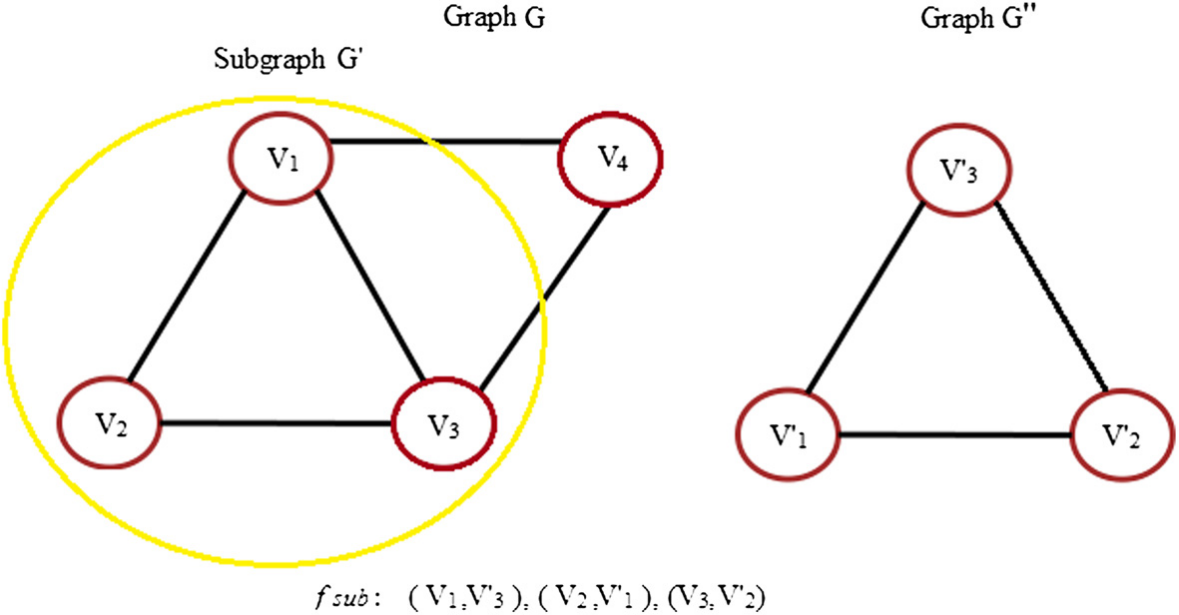
**Subgraph isomorphism.** An example of subgraph isomorphism between graphs G and G”, with highlighted graph G’ being a subraph of G. The subgraph G’ is isomorphic to G”.

**Figure 6:**
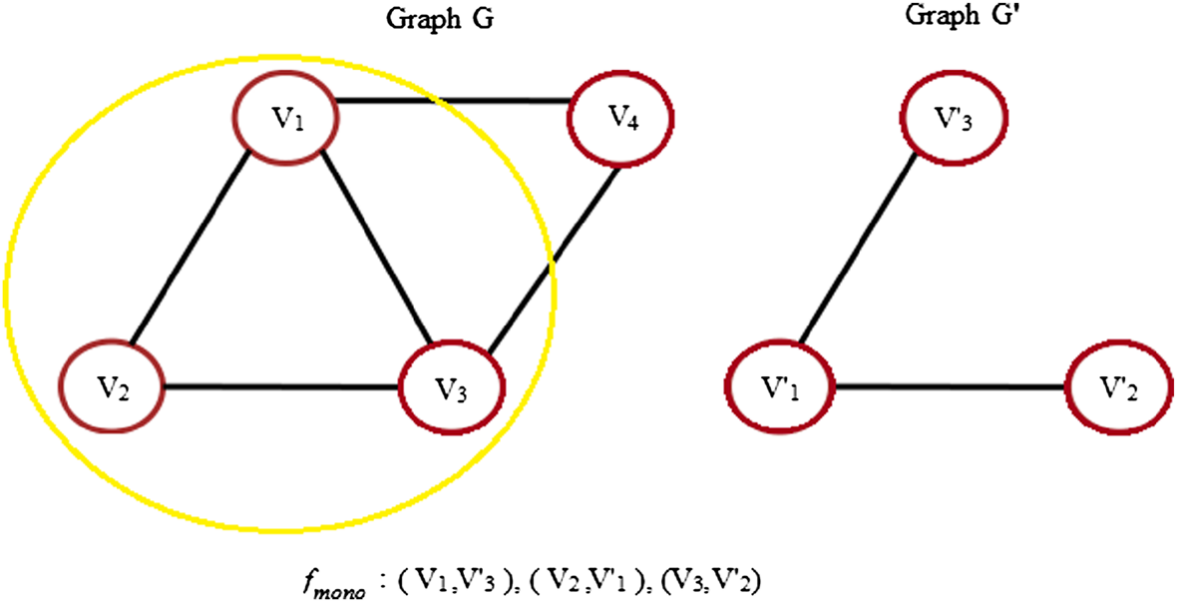
**Graph monomorphism.** An example of graph monomorphism between graphs G and G’.

Graph Matching is the process of comparing two graphs to find an appropriate correspondence between their nodes and edges. It refers to the process of finding a mapping *F*from the nodes of one graph *G*to the nodes of another graph *G*^*′*^ that satisfies some constraints or optimality criteria, ensuring that similar substructures in one graph are mapped to similar substructures in the other. Standard structural matching concepts include the following: 

1. **Graph Isomorphism:** It finds an exact structural correspondence between two graphs. It is a bijective mapping that preserves the number of nodes and edges. It is illustrated in Figure 4.

2. **Subgraph Isomorphism:** If nodes along with their corresponding edges are deleted from a graph *G*, a subgraph *G*^*′*^ denoted by *G*^*′ *^⊆ *G* is obtained. A subgraph isomorphism from *G* to *G*” is an isomorphism from a graph *G*” to a subgraph *G*^*′*^ of *G*. It is shown in Figure 5.

3. **Monomorphism:** It is a more relaxed matching than subgraph isomorphism as extra edges are also allowed between nodes in the larger graph. Figure 6 illustrates monomorphism from graph *G* to graph *G*^*′*^. Formally, it can be stated as: Let *G* and *G*’ be graphs. A graph monomorphism between *G*(*V*,*E*,*α*,*β*) and *G*^*′*^(*V*^*′*^,*E*,^*′*^*α*^*′*^,*β*^*′*^) is an injective mapping *F*_*mono*_:*V*→*V*^*′*^ such that:

(1) $$\alpha (v)=\alpha^{\prime }(F_{\mathrm{mono}}(v))\;\forall \;v\in \mathrm{V.}$$

For any edge *e*=(*u*,*v*) ∈ *E*, there is an edge *e*^*′*^=(*F*_*mono*_(*u*),*F*_*mono*_(*v*)) ∈ *E*^*′ *^such that *β*(*e*)=*β*^*′*^(*e*^*′*^).

**Figure 7:**
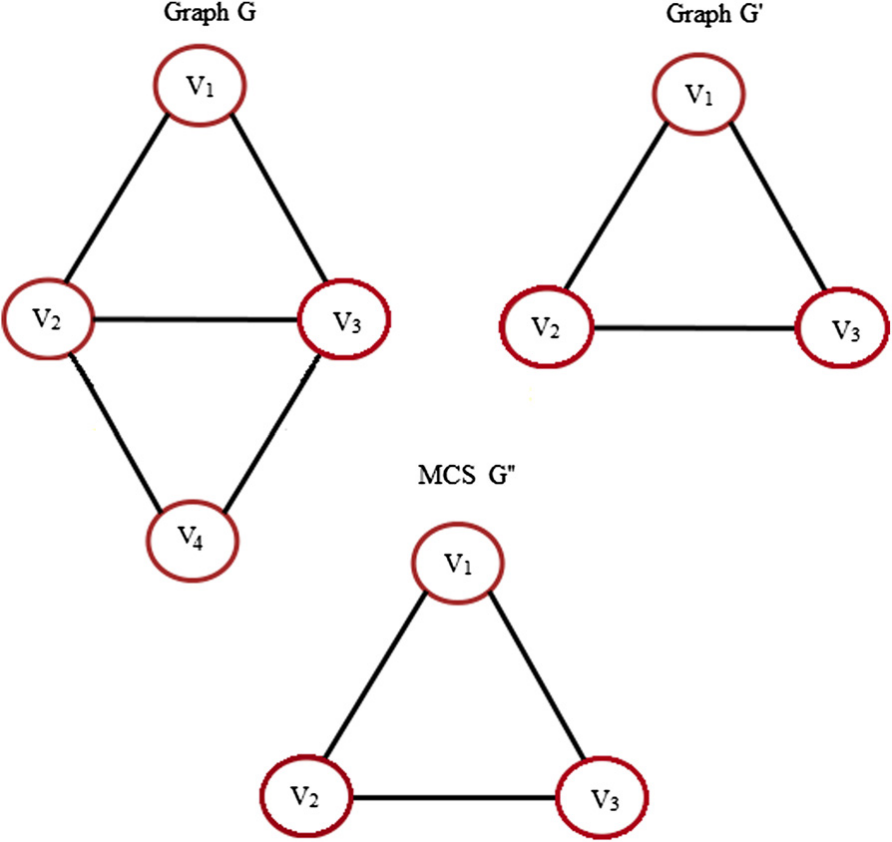
**Maximum Common Subgraph.** An example showing a MCS G” of graphs G and G’.

4. **Maximum Common Subgraph (MCS):** An MCS of two graphs, *G* and *G*^*′*^, is a graph *G*” that is a subgraph of both *G* and *G*^*′*^, such that it has the maximum number of nodes among all possible subgraphs of *G* and *G*^*′*^. MCS of two graphs is usually not unique. It can be used to measure the similarity of objects as the larger the MCS, higher will be the similarity. It is shown in Figure 7. A common subgraph of *G* and *G*^*′*^, *CS*(*G*,*G*^*′*^), is a graph *G*” such that there exist subgraph isomorphisms from *G* to *G*” and from *G*^*′*^ to *G*” or vice-versa. *G*” is a MCS of *G*and *G*^*′*^, *MCS*(*G*,*G*^*′*^), if it is the common subgraph with maximum nodes.

#### Types of graph matching

The two main types of graph matching are [23]: 

1. **Exact matching:** These methods find a strict correspondence between two graphs if it exists. Structurally, it ensures that the mapping between nodes of the two graphs must be ‘edge-preserving’, that means if two nodes in one graph are linked by an edge, they are mapped to two nodes in the other graph that are also linked by an edge. For ARGs, the matching ensures that the attributes are also identical in both graphs.

2. **Inexact matching:** The algorithms do not find a strict correspondence between two graphs but a more relaxed one, as there maybe a match between nodes where edges are not preserved. Further, for ARGs, the attributes of nodes and edges may differ. In this case, a cost (or distance) is calculated that takes into account differences among the corresponding attributes. The matching finds a mapping that minimizes this cost. It is used where the constraints imposed by exact matching are too strict for graphs used, such as graphs not identical to each other. Two types of inexact matching algorithms exist [24]: 

(a) Optimal inexact matching: These algorithms always find a solution that is the global minimum of the matching cost, i.e. they will find an exact solution if it exists. However, they are usually more expensive than exact ones as they require exponential time and space due to the NP completeness of the problem. Due to this reason, they are suitable for graphs with a small number of nodes and edges.

(b) Approximate or sub-optimal matching: These algorithms only ensure to find a local minimum of the matching cost. Not always ensured, but often the local minimum found is close to the global minimum. However, even if an exact solution exists, they may not be able to find it.

### The basic A* Search Algorithm

Dijkstra’s algorithm [25] starts with the source node and traverses the nodes in a graph such that shortest path from the source, found so far, is prolongated first. Thus, by reaching the goal node the shortest path is guaranteed to be found. On the other hand, the Greedy Best-First-Search algorithm [26] selects the node closest to the goal by using a heuristical estimate of the distance of a node from the goal node irrespective to distance to source and thus finds a path to the goal in shortest time, which is not necessarily the shortest path. A* algorithm [27] was developed to combine formal approaches like Dijkstra’s algorithm and heuristic approaches like Greedy Best-First-Search algorithm.

#### Path Scoring

A* finds the least-cost path in the graph from source node to goal node. To calculate the cost, it uses the following formula [27]:

(2) f(n)=g(n)+h(n)

where: 

• *g*(*n*) is the distance from source node to node *n*.

• *h*(*n*) is the heuristic function that is used as an estimate of the minimum cost from current node *n*to the goal node. It is important to choose a good heuristic function. The more accurate the heuristic the faster the goal node is reached and through shorter path.

• *f*(*n*) this is the current approximated cost of the shortest path to the goal node going through node *n*.

A* computes the sum *f*(*n*) of *g*(*n*) and *h*(*n*) as it moves from the source to the goal and selects the node with the lowest *f*(*n*) in each iteration. Let *h*^∗^(*n*) be the true minimal cost from *n*to goal. The behaviour of the algorithm depends on the heuristic *h*(*n*) as [28]: 

• If *h*(*n*)=0, then A* turns into Dijkstra’s algorithm as only *g*(*n*) plays a role. It is guaranteed to find a shortest path.

•If *h*(*n*) ≤ *h*^∗^(*n*), then A* is guaranteed to find a shortest path. The lower *h*(*n*) is, the more nodes are expanded, making it slower.

• If *h*(*n*)=*h*^∗^(*n*), then optimal path will be found and no other nodes will be expanded, making it very fast. Hence for a given perfect heuristic, A* will behave perfectly.

• If *h*(*n*) >*h*^∗^(*n*), then A* is not guaranteed to find the shortest path, but it can be even faster than the optimal *h*(*n*)=*h*^∗^(*n*) case.

• If *h*(*n*) ≫ *g*(*n*), then A* turns into Greedy Best-First-Search algorithm as only *h*(*n*) plays a role.

#### Implementation

**Figure 8:**
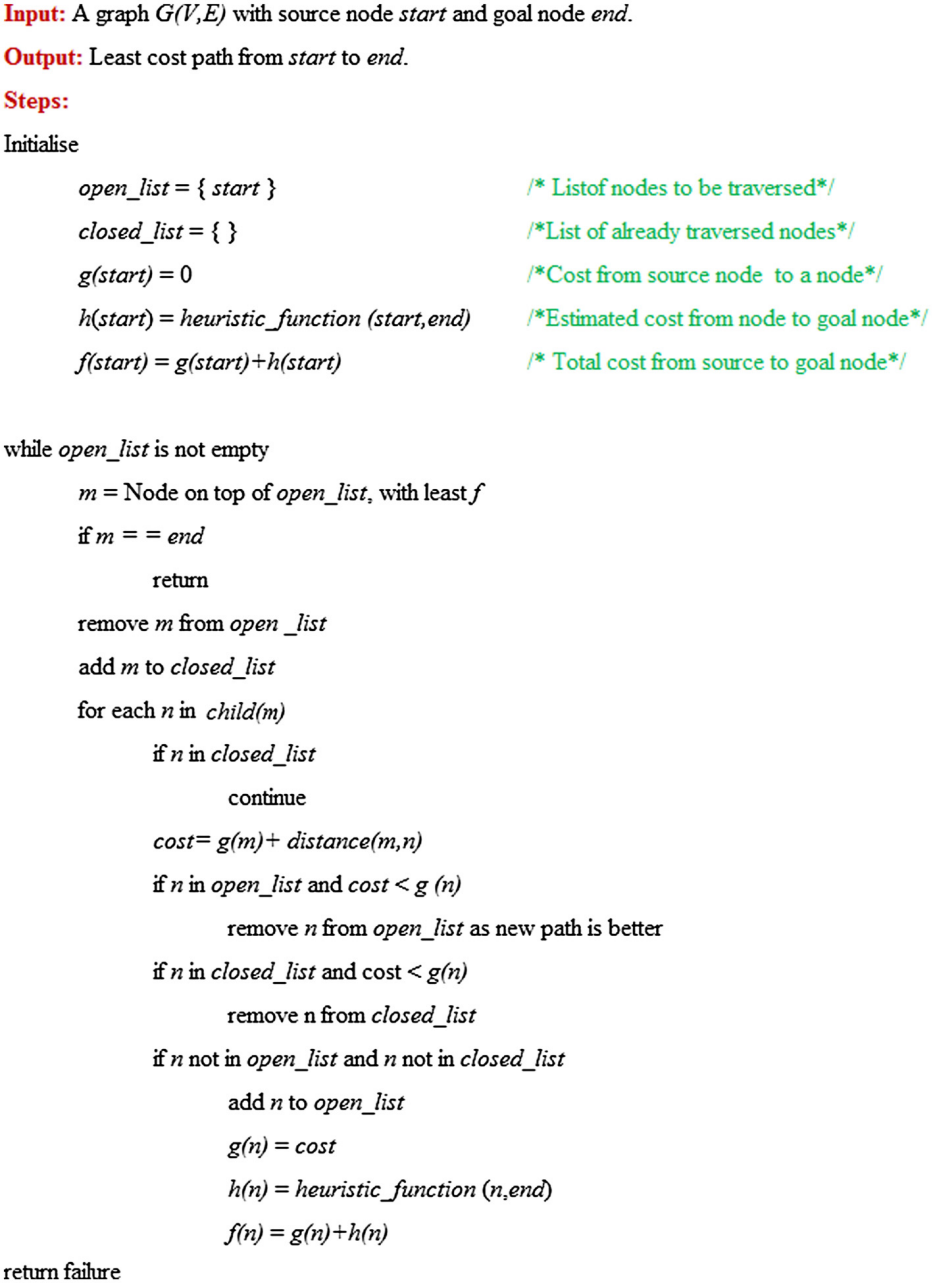
A* search algorithm. Pseudocode of the A* search algorithm operating with open and closed lists of nodes.

A* algorithm can be implemented by maintaining of two lists: the Open List and the Closed List. The Open List contains nodes that are candidates for examining. It is generally maintained as a priority queue, as the node with highest priority is the one with least *f*(*n*) cost. Initially, it contains just one element: the source node. The Closed List contains those nodes that have already been traversed and form the optimal path. At each step of the algorithm, the node *n* with the lowest *f*(*n*) value is examined from the Open List. If *n*is the goal, then algorithm stops. Otherwise, it is removed from Open List and added to Closed List, and the *f*(*n*), *g*(*n*) and *h*(*n*) values of its child nodes are updated accordingly. These nodes are then inserted into the Open List queue and are synchronised according to priorities of other existing elements. The process continues till the goal node is reached, or no more nodes are available in Open List (a case of no solution). The algorithm is shown in stepwise manner in Figure 8.

#### Properties

The properties of A* search algorithm are given as: 

1. **Completeness:** A* is complete, as it takes an input, evaluates the paths possible from source to goal, and returns a solution if it exists. Hence, if there is a solution, it will be found.

2. **Admissibility:** For optimal performance A* must be admissible, i.e., *h*(*n*) should be a lower bound on the true minimal cost *h*^∗^(*n*) ( *h*(*n*) ≤ *h*^∗^(*n*)∀*n*). Then it would find an optimal path from source to goal if it exists.

3. **Complexity:** The time complexity depends on the value of *h*(*n*). When *h*(*n*) is very small (in the worst case), the number of nodes traversed is exponential to the length of the shortest path. However, when the search space is a tree, which holds true in the case considered here, goal is a single node, and *h*(*n*) meets the condition that the error of *h*(*n*) does not grow faster than the logarithm of *h*^∗^(*n*),

(3) $$\left|h(n)-h^{*}(n)|=O(\log(h^{*}(n))\right)$$

then the number of nodes traversed become polynomial [26].

## Methods

**Figure 9:**
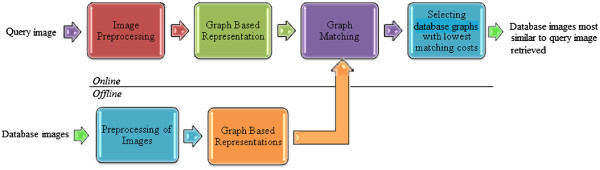
**Block diagram of the proposed method.** Schematic overview of the proposed CBIR method.

A block diagram of the method employed is given in Figure 9. The main steps are explained as: 

1. **Image acquisition:** H&E-stained breast biopsies are used in this study. Specimens are digitized and the whole-slide images (WSIs) are rescaled to about 100x effective magnification for further experimentation.

2. **Image segmentation:** In this step the images are prepared for graph-based description. It involves segmentation of the images as well as removal of artefacts and obtaining connected components in each segmented image.

3. **Graph-Theoretic representation:** The segmented images are then represented using ARGs which involve the description of nodes and edges.

4. **Graph matching:** The graph representing query image is then compared to a database of graphs already generated in order to retrieve most similar images based on the distance between the graphs. A graph matching algorithm based on the A* search is used.

5. **Display of the closest matches:** The images from the database are arranged in order of decreasing similarity based on cost of graph matching and the top results are displayed to the user.

### Image segmentation

It includes a group of methods employed before graph-based image analysis. To acquire a region-based signature, a key step is to segment images. Hence, the original breast biopsy images are first segmented using a supervised approach which has been performed in two stages: 

1. **Soft pixel classification:** Likelihood of belonging to a tissue of particular type is calculated for each image pixel based on texton-based texture descriptions.The segmentation decision is made for every point (local area) on MAP (Maximum A Posteriori) principle based on texture descriptions of all allowed tissue classes previously learned.

2. **Region segmentation:** Grouping of pixels and hard label assigment is performed based on spatial label coherence and similarity to texture models already obtained in the previous stage. Such optimal grouping is performed using Graph-cut [29] algorithm.

The maximum size of the pixel area for decision making is tissue type related. In these experiments 16× 16 pixels for epithelial, 32×32 pixels for connective, 48×48 for lobular and 64×64 for fat tissues were used. Here, the effective pixel size for these images (i.e. how large the physical area of the tissue which corresponds to one pixel) was roughly 1.0 micrometer ×1.0 micrometer. The segmentation algorithm is not described in further details here and is a subject to a separate publication. Segmentation results were just provided for this study. The segmentation is done into four tissue types: lobules, fibrous connective tissue, epithelial lining cells, lumens-&-fat (centres of ducts and adipose tissue).

The multilabel (L=4 here) segmented image is decomposed into binary images, one image for each label. Then morphological operations closing and opening are performed twice each on each binary image. These operations aim to remove small artefacts, fill in the potential gaps between tissue fragments and smooth the contours of the shapes. The size of structuring element chosen depends on the magnification of the WSIs used in the study. Then connected components are identified in each binary image. A connected component analysis ensures that only connected pixels are assigned the same label and form a region. It is required for distinguishing the regions within the image.

### Graph-theoretic Representation

Each of the images in the database as well as the query image have been described by corresponding graphs. Namely, ARGs have been constructed, where each node corresponds to one connected region in the image and edge is obtained between neighbouring regions which share a common boundary. The procedure involves describing nodes and edges with attributes explained below.

#### Node Description

Describing the nodes includes identifying the nodes and then assigning attributes to them. Each node has a unique identifier number that is used to simply recognise it in subsequent algorithm. Also, though a node denotes a region, for representational purpose, its position is assumed to be at the centroid of the region. The actual information about the region that each node carries is: 

• **Area:** It is defined as the total number of pixels inside the region corresponding to the node. The areas are found for each region, and regions with area of less than a predefined threshold are ignored and not considered as separate nodes.

• **Perimeter:** The attribute gives the length of the boundary of a region. It is computed by summing of distances between each adjacent pair of pixels along the border of the specified region.

• **Label:** It defines the class of tissue for the region.

Initially, other features were also considered for node attributes, however, they were not retained for the final implementation, since they were found unsuitable or inefficient for this particular application. Actually PCA would be the right way for selecting the appropriate attributes, however, in order to reduce the computational complexity, we have performed a heuristic selection of attributes. The features not retained for node description are: 

• **Convex area:** It is the number of pixels in the convex hull of a region.

• **Eccentricity:** For an ellipse, eccentricity is defined as the ratio between the distance between its foci and its major axis length. It has a value between 0 and 1. For a region, it is the eccentricity of the ellipse which has the same second-moments as that of the region.

• **Euler number:** It is defined as the difference between the number of objects in a region and the number of holes inside those objects.

• **Orientation:** It can be defined as the angle between the x-axis and the major axis of the ellipse which has the same second-moments as the region. Its value is between -90° to 90°.

• **Solidity:** It is the fraction of pixels in the convex hull that are also in the region and computed as ratio between the area of a region and its convex area.

#### Edge Description

The process of describing edges involves identifying the edges and assigning weights to them. The edge information (weights) is obtained as: 

• **Distance between centroids:** It is taken as the Euclidean distance between the centroids of two regions.

• **Common boundary length:** It is the number of pixels lying on the common border between two neighbouring regions. It has been calculated by considering the 4-connectivity of each pixel. The algorithm counts those 4-connected neighbours of a pixel which have a different label than the pixel itself.

**Figure 10:**
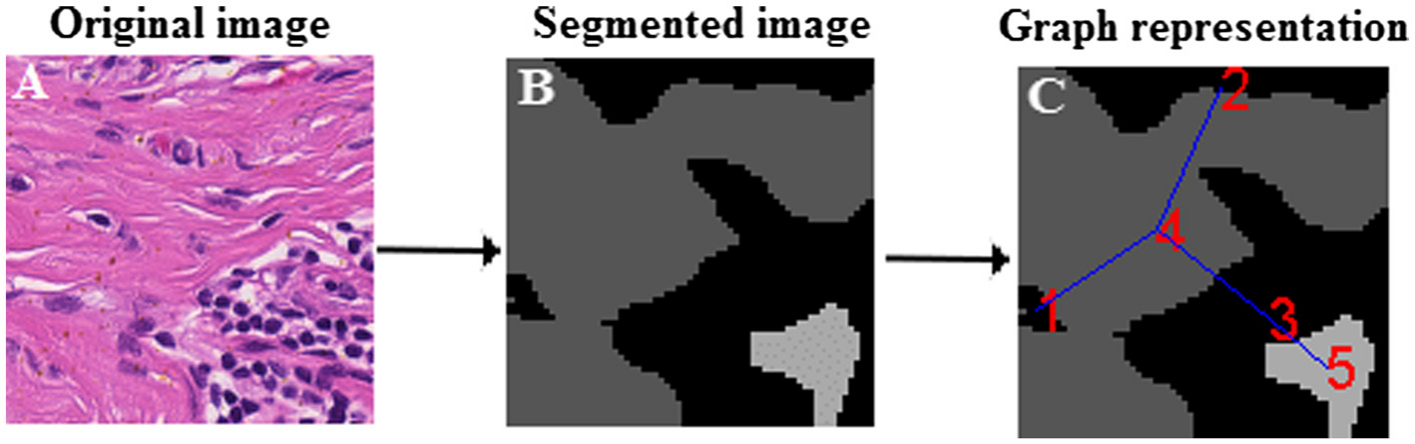
**Example of graph representation of histological image.** An example of graph-theoretic representation of a histological image. **A** shows a part of original histological image, **B** shows the segmented version of the image in **A** and **C** presents the graph obtained for the image (the obtained graph is overlaid on the image **B**).

Same as with nodes, other characteristics can also be included in edge attributes. However, it was found that the properties given above are suitable for representing a histological image. Area is important for matching of similarly-sized regions, whereas perimeter conveys approximate shape information. The distance between centers determines how far the nodes are placed with respect to each other, and common boundary length denotes upto what extent two regions are adjacent to each other. Thus, these properties are most useful for determining the structure and neighbourhood relationships for histological image analysis. One example of such a graph-representation is given in Figure 10.

#### Normalisation

The graph attributes obtained in the above steps are expressed in different units. Thus, this data need to be converted to relative units so that they becomes comparable in subsequent procedures. A global normalisation is performed. For each feature (except label of nodes), first the global maximum and minimum values are obtained from all the graphs in the database. Then the features are normalised to [0,1] range using the global maximum and minimum values for each one.

### A*-based graph matching method

Given a query image, its ARG is matched to each ARG in the database and the cost of matching is assigned to each pair of graphs. The graph matching problem has been formulated as an A* based tree search problem. Functions for the cost *g*(*n*), heuristic *h*(*n*) and total cost *f*(*n*) have been designed using the information present in the corresponding image ARGs. The heuristic *h*(*n*) is designed to be a consistent lower bound estimate of the exact cost, hence, admissibility criterion is satisfied that leads to the optimal solution.

To describe the process of matching, two ARGs are defined first: A Test ARG *G*(*V*,*E*,*α*,*β*) and Model ARG *G*^*′*^(*V*^*′*^,*E*,^*′*^*α*^*′*^,*β*^*′*^). *N* is the number of nodes in *G* and *N*^*′*^ is number of nodes in *G*^*′*^such that *N* ≤ *N*^*′*^. Also, *W* is the number of edges in *G* and *W*^*′*^ is the number of edges in *G*^*′*^. For each node *n*_*i*_(*i* ∈ 1,2…*N*) in *G*, the set of attributes is given as *a*_*i*_:

(4) $$a_{i}=a_{i}^{\left(1\right)},a_{i}^{\left(2\right)}\mathrm{..}a_{i}^{\left(k\right)}\mathrm{...}a_{i}^{\left(K\right)},\;\;\;k\in \{1,2\mathrm{..K}\}$$

where *K* is the total number of attributes associated with each node *n*_*i*_. Hence, for all nodes *N*, the set **a** is the set of all vectors **a**_**i**_ given by:

(5) $$a=a_{1},a_{2},a_{3},\mathrm{...}a_{N}$$

Similarly, for each edge *e*_*j*_(*j* ∈ 1,2…*W*) in *G*, the set of attributes is given as *b*_*j*_:

(6) $$b_{j}=b_{j}^{\left(1\right)},b_{j}^{\left(2\right)}\mathrm{..}b_{j}^{\left(m\right)}\mathrm{..}b_{j}^{\left(M\right)},m\in \{1,2\mathrm{..M}\}$$

where *M* is the total number of attributes associated with each edge *e*_*j*_. Hence, for all edges *W*, the set **b** is the set of all vectors **b**_**j**_ given by:

(7) $$b=b_{1},b_{2},b_{3},\mathrm{...}b_{W}$$

For the proposed method, *K*and *M*both have value 2, where k=1 for area and k=2 for perimeter in node attributes, and m=1 for distance between centroids and m=2 for common boundary length in edge attributes. Note that for node attributes, area has been assigned double the weight of perimeter, as it is considered more important feature during the matching of nodes.

The task is to find the best mapping between *G*and *G*^*′*^ and the minimum matching cost for attaining it. A graph monomorphism is being sought between *G* and *G*^*′*^ as explained in Section Graph Matching. To begin with, the simplest case can be to assign the number of unmatched nodes as the heuristic function that denotes estimate of cost of a path from a node to goal, and the number of matched nodes as the cost function that denotes the cost from start to a node. For a partial mapping till node *n* in *G*(*n* ≤ *N*), these functions will be defined as:

(8) g(n)=n

(9) h(n)=N−n

The dissimilarity of the nodes (and correspondent edges) must also be incorporated in these formulations. A pairwise distance between feature vectors of nodes already matched needs to be included in equation 8, and the term containing attributes for unmatched nodes should be included in equation 9. It gives:

(10) $$\begin{array}{llll} \;g(n)=\overset{n}{\underset{i=1}{\sum }}\delta_{i}+n & & & \end{array}$$

$$\begin{array}{llll} \;=\overset{n}{\underset{i=1}{\sum }}(\delta_{i}+1) & & & \end{array}$$

(11) $$\begin{array}{llll} h(n)=\overset{N}{\underset{i=n}{\sum }}a_{i}^{\left(1\right)}+(N-n) & & & \end{array}$$

$$\begin{array}{llll} \;=\overset{N}{\underset{i=n}{\sum }}(a_{i}^{\left(1\right)}+1) & & & \end{array}$$

where, *δ*_*i*_ in equation 10 describes the distance between the attributes of already matched nodes and edges, and $a_{i}^{\left(1\right)}$ in equation 11 refers to areas of the nodes in *G*not matched yet. Note that only the area attribute is used at this point as computing *δ*_*i*_ will not be possible for unmatched nodes and the most important attribute that needs to be considered in the heuristic function is area.

Now, let us consider two incremental functions *g*_*Δ*_(*n*) and *h*_*Δ*_(*n*) which denote the contribution of a node *n*, to the cost function *g*(*n*), if it has been already traversed, and its contribution to the heuristic *h*(*n*) if it has not been already traversed. These functions can be defined as:

(12) $$\begin{array}{llll} g_{\Delta }(n)=g(n)-g(n-1) & & & \end{array}$$

(13) $$\begin{array}{llll} h_{\Delta }(n)=h(n)-h(n+1) & & & \end{array}$$

From the equations 10 and 11, it follows that:

(14) $$\begin{array}{llll} g_{\Delta }(n)=\delta_{n}+1 & & & \end{array}$$

(15) $$\begin{array}{llll} h_{\Delta }(n)=a_{n}^{\left(1\right)}+1 & & & \end{array}$$

The constant 1 in these equations must be re-scaled, otherwise it will have a greater impact and may mask the effect of attributes of ARGs. For this reason, a constant c is introduced, which has been determined experimentally. After this the equations become:

(16) $$\begin{array}{llll} g_{\Delta }(n)=\delta_{n}+c & & & \end{array}$$

(17) $$\begin{array}{llll} h_{\Delta }(n)=a_{n}^{\left(1\right)}+c & & & \end{array}$$

In order to yield optimum results, the admissibility criterion must be satisfied, i.e. the estimated cost of a path from a node to goal must be a lower bound of the actual cost of a path from the node to goal. This can be ensured if the estimated cost contributed by *n*, represented by *h*_*Δ*_(*n*) is by *n*, represented by *g*_*Δ*_(*n*). As *δ*_*n*_ ≥ 0 and $a_{n}^{\left(1\right)}\in [0,1]$, remember that all attributes have been normalized to the range [0,1] aforehand, in order to ensure that *h*_*Δ*_(*n*) ≤ *g*_*Δ*_(*n*) a constant 1 is added to the distance *δ*_*n*_. It now yields:

(18) $$g_{\Delta }(n)=(\delta_{n}+1)+c$$

The problem that may arise here is that as 1 becomes very large compared to distances *δ*_*i*_, the effect of distance may be masked, hence, a new constant, *γ*, is introduced. It is an experimentally determined parameter and distance becomes:

(19) $$d_{n}=\gamma \cdot \delta_{n}$$

Rewriting the equations 10 and 11 for the nodes traversed up to node *n*, *g*(*n*) and *h*(*n*) take the form:

(20) $$g(n)=\overset{n}{\underset{i=1}{\sum }}(d_{i}+1)+n\cdot c$$

(21) $$h(n)=\overset{N}{\underset{i=n}{\sum }}a_{i}^{\left(1\right)}+(N-n)\cdot c$$

The equation 20 can be used for *g*(*n*), however, for histological images, it is important that larger nodes are given higher importance in matching. This is because smaller nodes may represent less important regions or artefacts, but the larger nodes will always represent significant regions. A mismatch between larger nodes should be penalised by a higher cost as compared to mismatch between smaller nodes. Hence, a weight has been introduced:

(22) $$g(n)=\overset{n}{\underset{i=1}{\sum }}w_{i}(d_{i}+1)+n\cdot c$$

The weights *w*_*i*_ are proposed as:

(23) $$w_{i}=\max({a_{p}}^{\left(1\right)},{a_{q}^{\prime }}^{\left(1\right)})$$

where *n*_*p*_ ∈ *G* and $n_{q}^{\prime }\in G^{\prime }$ are matching nodes and *a*_*p*_^(1)^ and ${a_{q}^{\prime }}^{\left(1\right)}$ denote the first attributes corresponding to feature vectors **a**_**p**_ and $a_{q}^{\prime }$. They describe the area of the two regions being matched.

The distances *δ*_*i*_ in equation 10 between each pair of nodes are calculated as:

(24) $$\delta_{i}=\lambda \delta_{1i}+(1-\lambda )\delta_{2i}$$

where, *δ*_1*i*_ is the distance between corresponding nodal attributes and *δ*_2*i*_ is the distance between their corresponding edge attributes. *λ*∈[0,1] balances the mutual relevance of the two distances. In the method, equal relevance has been considered. The distance between nodes and edges has been formulated as an Exponential distance, in order to further intensify the mismatch between corresponding attributes, as compared to a linear technique or Euclidean distance. The nodal distance for node attributes is defined as:

(25) $$\delta_{1i}=\overset{K}{\underset{k=1}{\sum }}e^{\left|{a_{p}}^{\left(k\right)}-{a_{q}^{\prime }}^{\left(k\right)}\right|}\;-K,\;\;\;\;k\in \{1,2\mathrm{..K}\}$$

Edge distance for edge weights is defined as:

(26) $$\delta_{2i}=\overset{M}{\underset{m=1}{\sum }}e^{{|b_{p}}^{\left(m\right)}-{b_{q}^{\prime }}^{\left(m\right)}|}\;-M,\;\;\;\;m\in \{1,2\mathrm{..M}\}$$

The total cost *f*(*n*) of the partial mapping till node *n*is given by:

(27) f(n)=g(n)+h(n)

**Figure 11:**
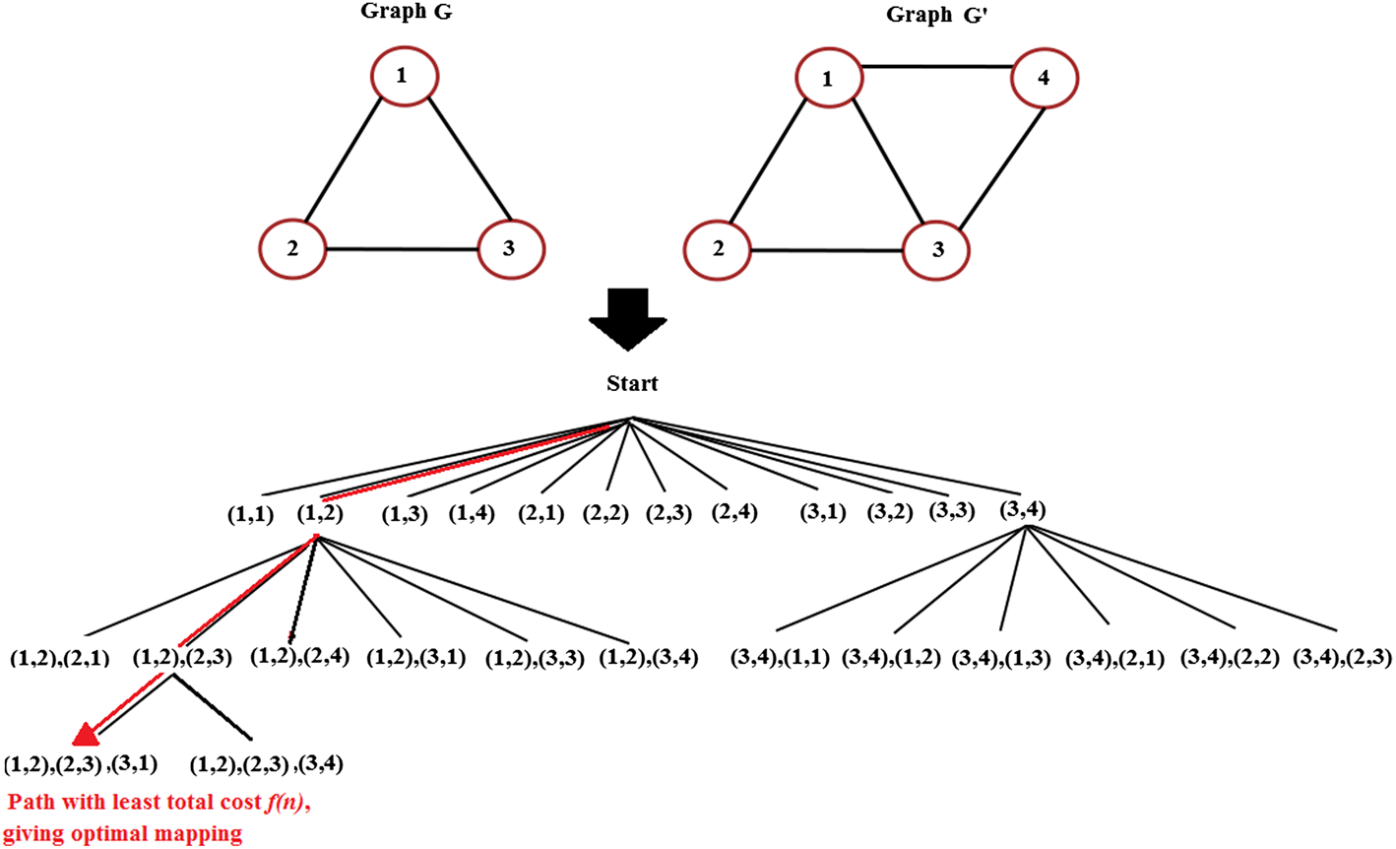
**Example of tree-based graph matching process.** An example of the search tree traversed by the A* algorithm during the matching of two graphs G and G’.

The graph matching is implemented through a tree-based search using A* algorithm which always extends the partial mapping of nodes towards an optimum. The tree is the representation of Open List realized using a priority queue, containing partial mappings in increasing order of their costs. It is constructed by first allowing each test node to be mapped to each available model node, if the match is permissible, the pairs forming the first level of the tree. The cost of each pair is computed, and the pair with the lowest total cost *f*(*n*) is expanded. Each leaf of the tree now represents a combination of matched nodes or partial mapping from nodes of test graph to those of model graph. The Closed List consists of the latest and most favourable partial mapping constructed. The tree is expanded until best optimum mapping of maximum nodes is found. An example of the tree-based search method employed is illustrated in Figure 11.

The main problem with optimal graph matching is its high computational complexity. The complexity of the described search is exponential in the worst case, however, practically, it depends on the data to be handled as only nodes of same label can be matched. It considerably reduces the search space and complexity is scaled down.

## Results and Discussions

### Dataset

The data used for this work consists of histological images provided by The Charité Hospital, Berlin. These are biopsy images of the breast tissue. The samples have been stained with the H&E dye. The WSI images are produced by a Zeiss MIRAX SCAN WSI scanner. We used selected archived slides from daily clinical workload that were not older than 6 months at the time of digitalization. The glass slides have been produced in AP-laboratory of the Institute of Pathology at Charité hospital. They have not been modified in any way. We have evaluated the method on 3 WSI images of FEA-suspected breast biopsies divided into sub-images representing possible retrieval results. Our aim was to demonstrate the potential of the graph-based approach, leaving the in-depth performance evaluation for future research. One of the reasons for this is the relatively high computational complexities of the segmentation, the description and the retrieval algorithm, which are subject for future change and improvements too.

The images have been pre-segmented to four categories describing different types of tissue. They are then divided for one approach (described in Section Experimental Approaches and Results) into smaller sub-images to obtain the database for different image sizes of 64×64, 128×128, 256×256 and 512×512. Query image is selected by giving a choice of four sizes, and the selection is resized to the size selected by the user. The number of images used in the database, depending on the size of query image is: 

64×64**:** 70869 images

128×128**:** 27596 images

256×256**:** 9132 images

512×512**:** 2485 images

Graph representations of the images stated above were obtained and stored for future reference.

### Experimental Approaches and Results

Two types of configurations were used for experiments. These are as follows:

#### Subgraph isomorphism approach

**Figure 12:**
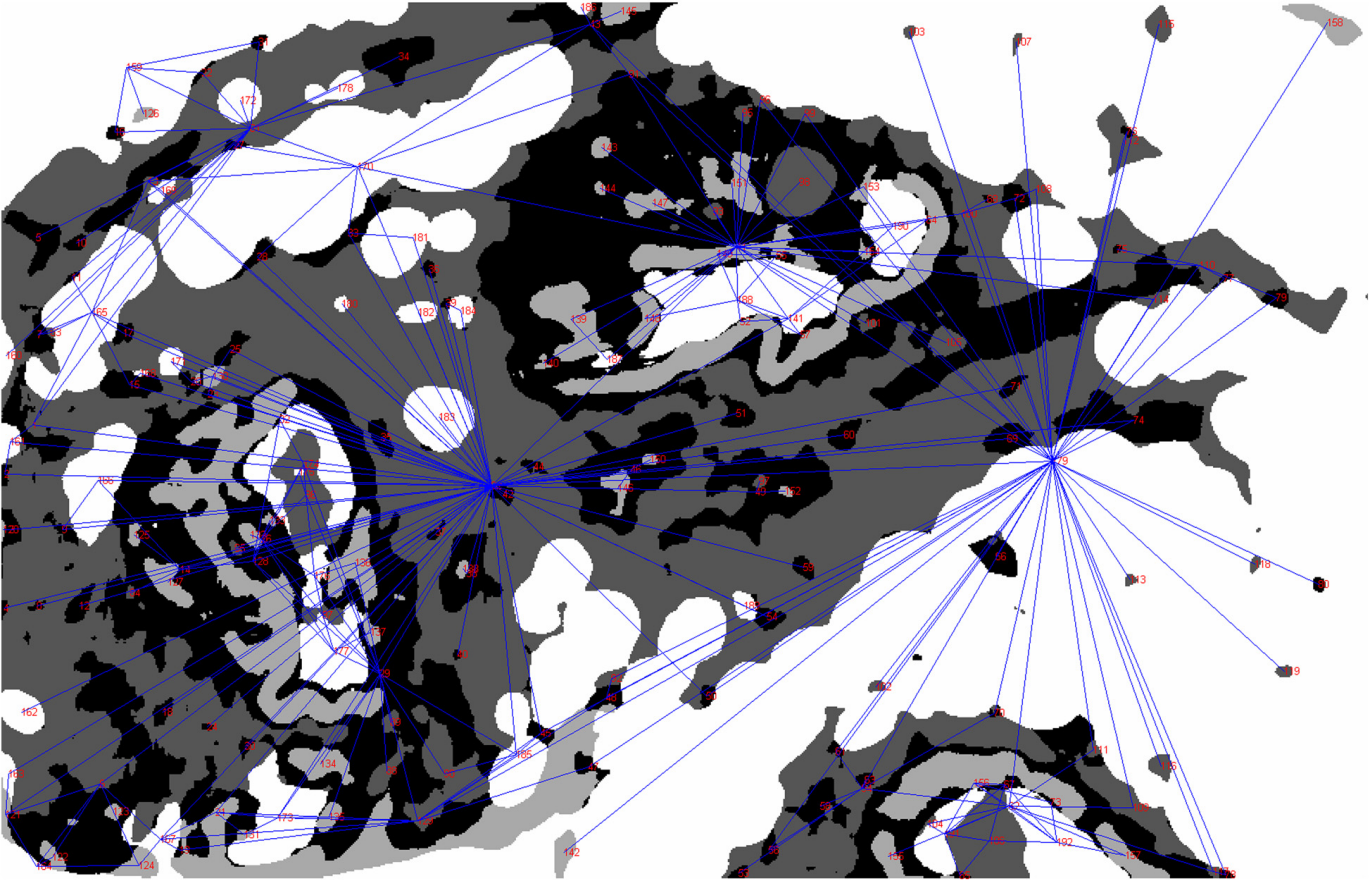
**Graph-based representation of histological image.** Graph obtained for one histological image from our database, overlaid on the correspondent segmented histological image.

**Figure 13:**
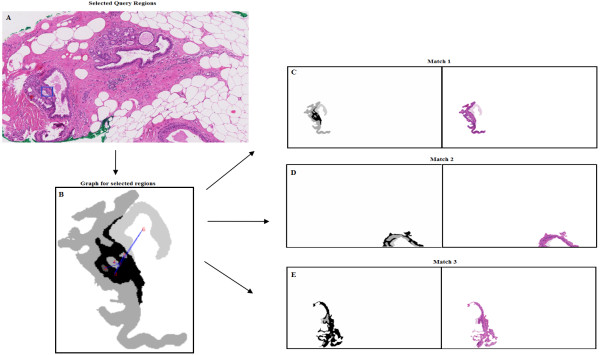
**Result example for first, subgraph isomorphism approach.** It is an example of the result obtained for subgraph isomorphism approach. The query selected, obtained RAG and three nearest matching results obtained are shown. In **A** the selected query image inside the whole image is depicted by blue rectangle. **B** shows the extended regions, selected by the query, and the corresponding graph formed for the selection. **C**, **D** and **E** give the first three results retrieved by the graph matching algorithm (both segmented and coloured).

The approach aims to find the subgraph isomorphism between a smaller query graph and the graphs obtained for the whole size images. The user submits a query image of any size by selecting a rectangular section of the whole histological image presented to him. The group of pre-segmented regions present in the selection is identified. Each of the regions is then extended to its original size and shape. An RAG is then constructed whose attributes are computed from the properties and spatial information of the extended regions. The matching process returns those subgraphs of the graphs obtained from the database of entire WSIs, which are closest to the graph obtained for the query. An example graph, generated for an entire histological image, is shown in Figure 12. The selection of the query image, the obtained RAG and three nearest matching results are shown in Figure 13. Here, a query consisting of lobular cells surrounded by epithelium and adjacent to a layer of fat tissue is selected. The closest matches show similar structural groups of lobules, fat cells and epithelial cells.

#### Inexact graph matching approach

**Figure 14:**
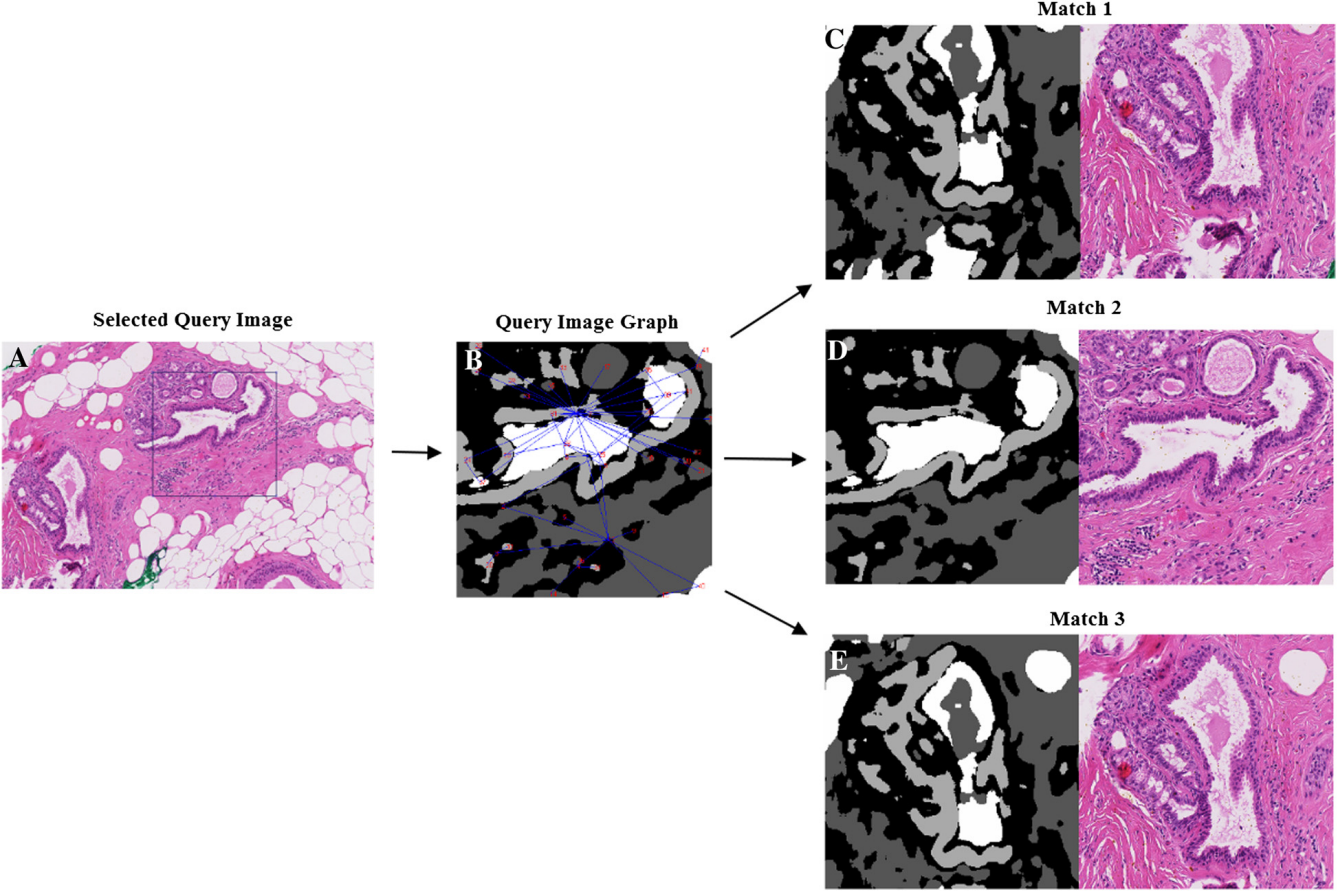
**Result example for second, inexact graph matching approach.** It shows an example of the result obtained for the inexact graph matching approach. **A** shows the query image selected from the whole image. The selection rectangle was fixed to 512x512 pixels. **B** depicts the graph formed for the query image. **C**, **D** and **E** give the first three results retrieved by the graph matching algorithm (both segmented and coloured).

In this approach, an inexact matching between a pair of graphs generated for a query image and a database image of the same size is determined. For this, first the database images are divided into smaller sub-images, query image is selected of a predefined size, and then the graphs of each sub-image is compared with the graph of the query image. The sub-images with closest similarity are retrieved. Note that in contrast to the previous method the regions of the query image are not extended to their original size. An example of this approach with results shown for a query size of 512×512 is shown in Figure 14. The query image has a duct with a lumen (center) with an outer lining of epithelial tissues and having lobules and connective tissue in the background. The retrieved results show similar regions. First result is not exact but depicts similar physiological structures and spatial relationships between them. Moreover, although the first and the third match basically show the same histological structure, due to the splitting of the whole histological image into sub-images they appear as different images in our database. They both have been selected due to the high similarity to the query image.

### Observations

It can be observed in both approaches that:

#### For subgraph isomorphism approach

The method yields regions from the whole images which are closest matches to the region in the query image. 

**Advantage:** As expected, the first match gives an exact match, as the graph generated is a subgraph from the graph of one of the whole images. The results obtained as subsequent matches show similarity in the structure and spatial relationships between regions, as those in query image. Hence, it can be used to locate region groups with desired type, shape and neighbourhood relationships.

**Limitation:** It does not take into account the size of query image. It considers all the regions which are present in the query image, including those regions which are only partially included in the query, and may have a large part outside the query. As a result, the matches obtained have the size corresponding to entire regions rather than the size of query. Hence, there is no control on the size of retrieved results.

#### For inexact graph matching approach

In this method, sub-images which are structurally similar to the query image, and of same size as query image, are retrieved. It works similar to a practical CBIR system. 

**Advantages:** The user can select a size for his query and the retrieved images are of same size as query. Hence the user can enjoy control on the size of results. The matches obtained are observed to show spatial and structural similarity to the query selected.

**Limitations:** Selection of query image may not be in accordance as the division of images into sub-images, and this may lead to truncation effect, as some important structures maybe truncated due to this division. Further, the original images have to be first cropped to a size which is divisible by the size of sub-image, and this can also lead to loss of information along borders. In order to reduce this information loss, the division of WSI images has been done by allowing an overlap between successive sub-images. However, if we increase the overlap, there is an increase in redundancy of results, as they may be retrieved from same areas. As a result there is a trade-off between redundancy and loss of information due to truncation, and overlap selected has to balance this.

### Performance evaluation

Evaluation is a crucial aspect for CBIR related areas. In this work, subjective evaluation has been performed as no known objective method was found appropriate. It is important to note that the evaluation has been performed only by a single observer, and the rates obtained are dependent on subjectivity and interpretation. It is a well known fact that precision and recall are the most popular measures used for evaluation of CBIR systems. They are defined as: 

1. **Precision:** The percentage of retrieved images that are relevant to the query:

(28) $$\mathrm{Precision}=\frac{\mathrm{Number}\;\mathrm{of}\;\mathrm{relevant}\;\mathrm{images}\;\mathrm{retrieved}}{\mathrm{Total}\;\mathrm{number}\;\mathrm{of}\;\mathrm{images}\;\mathrm{retrieved}}\times 100$$

2. **Recall:** The percentage of all the relevant images in the search database which are retrieved, defined by:

(29) $$\mathrm{Recall}=\frac{\mathrm{Number}\;\mathrm{of}\;\mathrm{relevant}\;\mathrm{images}\;\mathrm{retrieved}}{\mathrm{Total}\;\mathrm{number}\;\mathrm{of}\;\mathrm{relevant}\;\mathrm{images}}\times 100$$

There is no ground-truth available for the histological images, and assessment is performed purely by subjective analysis. Therefore, it is not possible to determine the total number of relevant images in a database, with respect to query due to which calculation of recall can not be performed. To demonstrate effectiveness of the method, precision is measured at scope lengths 10, 20, 30, 40 and 50. The scope length is the number of retrieved images. Precision for different scope lengths is calculated as:

(30) $$P_{s}=\frac{\overset{s}{\underset{i=1}{\sum }}\mathrm{scor}e_{i}}{s}\times 100$$

where *P*_*s*_ is precision (in %), *s*is scope length and *score* is a value from {0,0.25,0.5,0.75,1}. The score values express the similarity of the retrieved images to the query image in terms of structure and spatial relationships. Higher score is assigned for higher resemblance. The evaluation is subjective and coarse, so quantitative results (precision values) have been rounded down to integers. Resulted plots are plotted between the precision vs. different scope lengths.

The proposed method has been compared with a common, histogram-based retrieval system. The histograms for segmented sub-images have been found using 4 bins. Then the distances between the histograms of query image and sub-images have been calculated. Similar as for the graph-based approach, exponential distance has been used. Finally, the results were compared for both methods.

**Figure 15:**
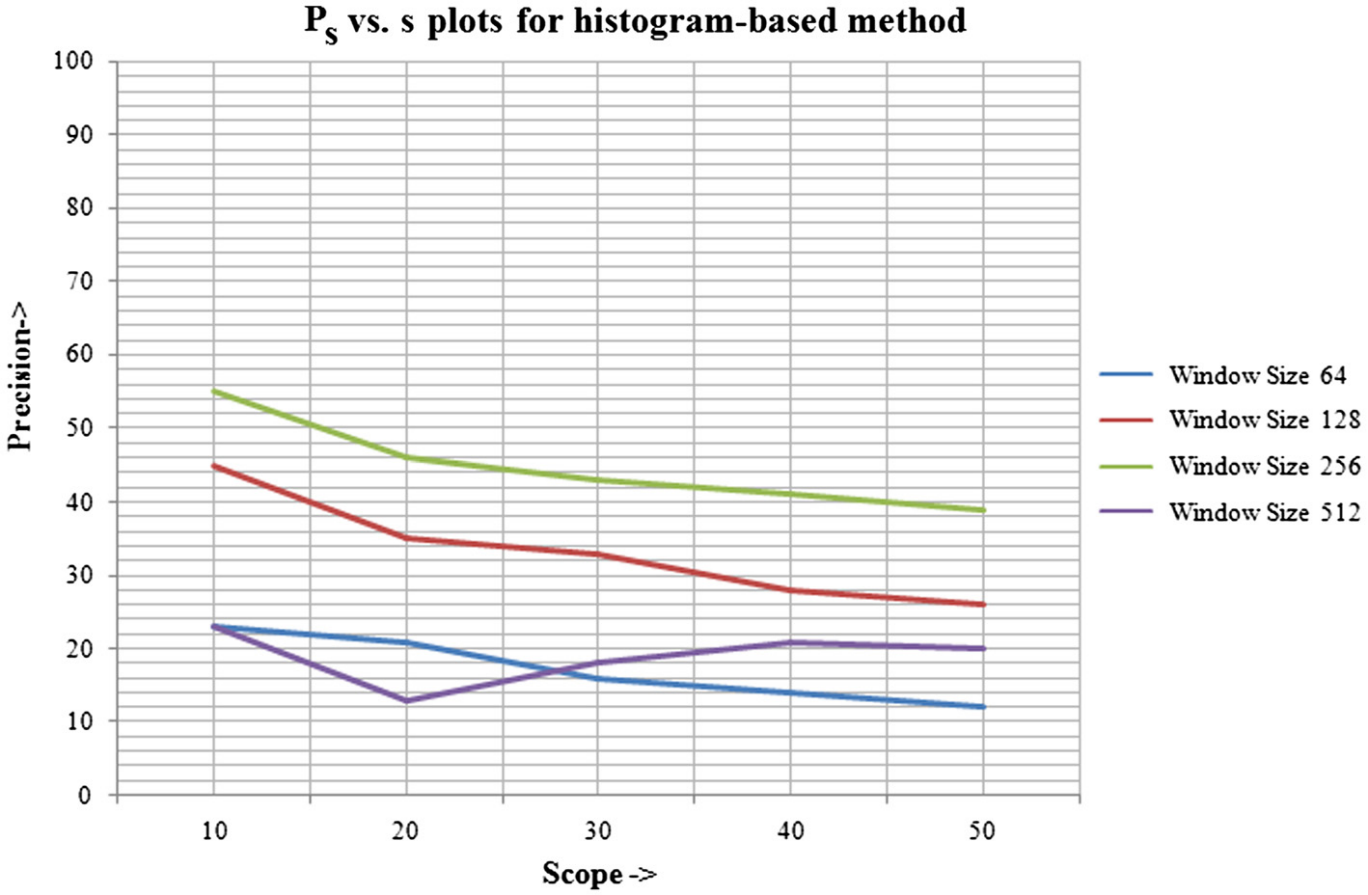
*****P***_***s ***_*vs. s*plots for histogram based method.** The precision vs. scope length plots for different window sizes for the histogram based approach are given in this figure. This evaluation refers to the second, inexact graph matching approach.

**Figure 16:**
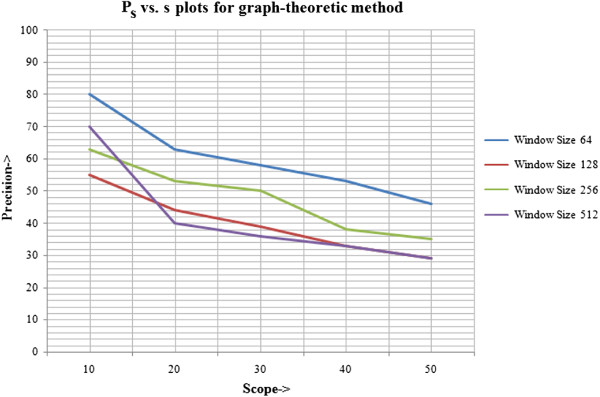
*****P***_***s ***_*vs. s*plots for graph-theoretic method.** The precision vs. scope length plots for different window sizes for the graph-theoretic approach are given in this figure. This evaluation refers to the second, inexact graph matching approach.

**Figure 17:**
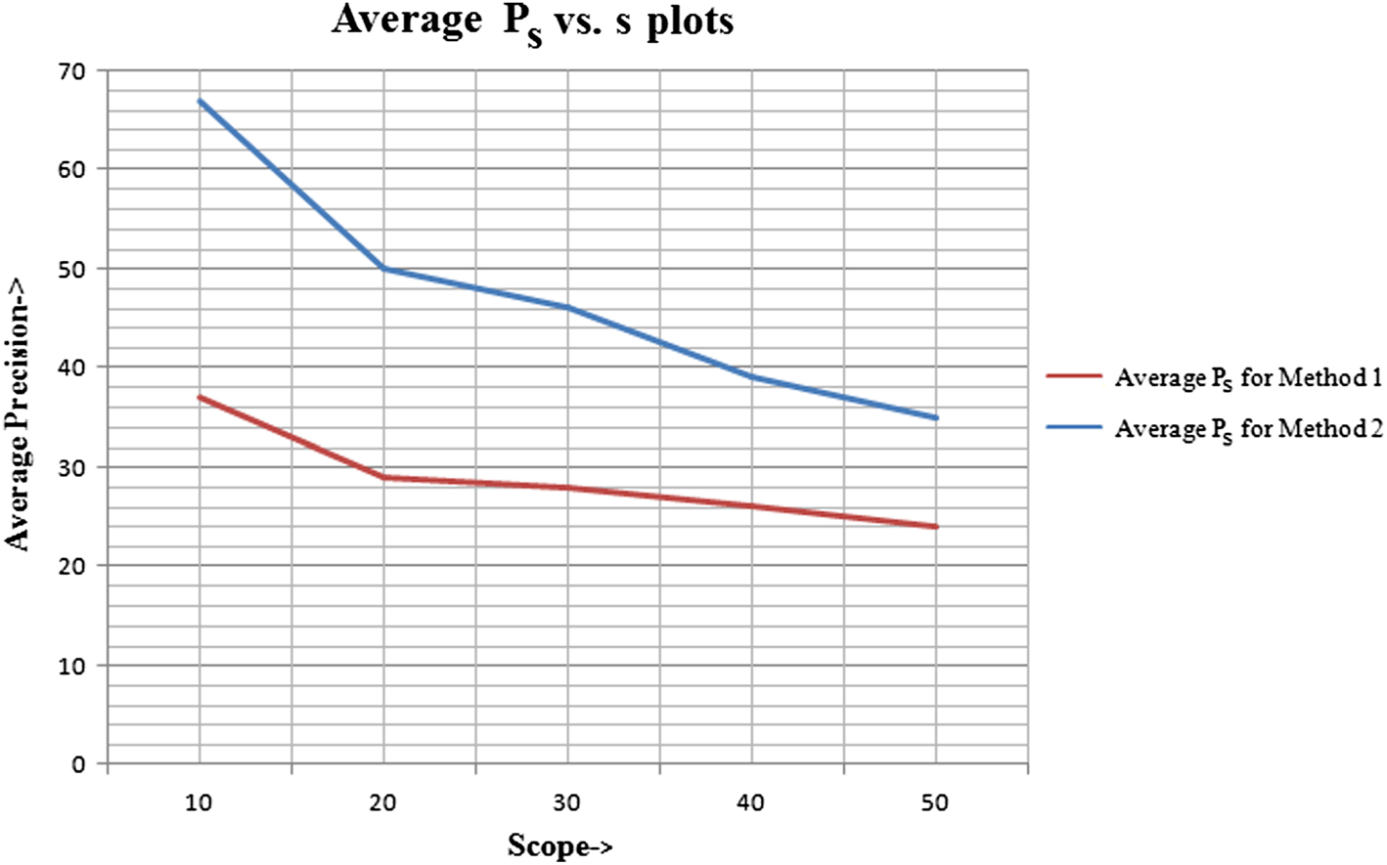
**Average ***P***_***s ***_*vs. s*plots for both, the histogram and the graph-theoretic, methods.** The average precision vs. scope length plots, across all window sizes, for both, the histogram (Method 1) and the graph-theoretic (Method 2) methods are given in this figure.

For the histogram-based technique, the table of precision for different scope lengths at different window sizes (or query sizes) is given in Table 1, with corresponding line plots illustrated in Figure 15. The table of precision for scope lengths at the same query sizes and the line plots for graph-theoretic method are shown in Table 2 and Figure 16, respectively. Next, a comparison is established between the average precision of both methods, calculated across all window sizes. The relative improvement of graph-based technique over histogram-based technique is given in Table 3, with corresponding line plots visualised in Figure 17.

**Table 1:** Precision at different scope lengths for histogram based method

**Precision at different scopes for histogram based method**					
***P*_*s*_/ Window size**	**64×64**	**128×128**	**256×256**	**512×512**	**Average *P*_*s*_**
*P*_10_	23	45	55	23	**37**
*P*_20_	21	35	46	13	**29**
*P*_30_	16	33	43	18	**28**
*P*_40_	14	28	41	21	**26**
*P*_50_	12	26	39	20	**24**

**Table 2:** Precision at different scope lengths for graph-theoretic method

**Precision at different scopes for graph-theoretic method**					
***P*_*s*_/ Window size**	**64×64**	**128×128**	**256×256**	**512×512**	**Average *P*_*s*_**
*P*_10_	80	55	63	70	**67**
*P*_20_	63	44	53	40	**50**
*P*_30_	58	39	50	36	**46**
*P*_40_	53	33	38	33	**39**
*P*_50_	46	29	35	29	**35**

**Table 3:** Average precision for both, the histogram and the graph-theoretic methods

**Average precision for both CBIR methods**			
**Scope length**	**Average *P*_*s*_ for Method 1**	**Average *P*_*s*_ for Method 2**	**Improvement (%)**
10	37	67	**81**
20	29	50	**72**
30	28	46	**64**
40	26	39	**50**
50	24	35	**46**

Method 1 denotes the histogram based method and Method 2 denotes the graph-theoretic method.

The tables and graphs obtained justify our choices of the parameters used and methods employed for the system proposed. It can be concluded that: 

The results obtained using graph-theoretic technique are better than simple histogram based method, as it takes into account the structural characteristics of the image and neighbourhood relationships between regions, which are completely neglected in the histogram-based method.

As scope length increases, the precision declines for proposed method, which shows that it gives the most relevant results earlier in the list of retrieved results. This is a desirable property of any CBIR system that the results initially obtained are the most useful. However, it is evident that it does not hold for all cases of histogram-based method.

The results so obtained by the proposed method are not as high as reported for general CBIR applications (about 90% precision or more). The highest precision reported for image size 64×64 and scope length 10 is 80%. The reason behind this is the complexity and subjectivity associated with histological images. The evaluation was biased strongly with the subjective scoring as even when the histological image shows the same tissue composition, several factors have to be kept in mind before assigning a score. The closeness to query image depends on the type of tissue regions, size and shape of regions as well as the neighbourhood relationships between them. Due to this relative scores have been used, however, more thorough evaluation should be performed especially by employing medical professionals.

The performance depends on the characteristics of the query image, i.e. the number of tiled images available in the database that lie close to the position of the query window.

### Execution Time Requirements

It can be said that optimizing the execution time has not been a primary concern of the research. The execution time required for the whole process depends on the following factors: 

1. **Complexity of the images:** The time required for graph generation and graph matching is highly dependent on the complexity of database and query images, i.e. the number of nodes and edges.

2. **Size of database and query images:** Given the same overall complexity, for a larger sized query, more time is required, specially for matching. Nevertheless, for less complex larger images, the method gives quicker results when compared to more complex, but smaller images.

The approximate time required for the execution of the main procedures for graphs of different number of nodes are mentioned in Table 4. For other supporting methods, the time requirement is negligible, hence, not mentioned. It can be observed that, the method requires lesser execution time for smaller number of nodes. With reference to histological images used, it can be said that queries up to size 256×256 can be used with less significant time requirements. With larger query images, execution time can become a greater concern.

**Table 4:** Time requirement for graph based CBIR system

**Time requirement for graph based CBIR system**		
**Number of nodes**	**Graph Generation Time**	**Graph Matching Time**
& 5	&1 s	& 0.1 s (MATLAB)
5-10	1-2 s	0.1-1 s (MATLAB)
10-20	2-3 s	15-30 s (MATLAB)
20-50	3-20 s	15-30 s (C++)
50-100	20-40 s	60-300 s (C++)
>100	> 40 s	>300 s

The task of graph matching was performed using MATLAB for simpler graphs, and C++ for complex graphs, obtaining better execution times. The experiments were performed on AMD Phenom (tm) X4 945 Processor at 3.00 GHz with 4 GB RAM.

## Conclusions

In this work we have developed a novel method for determining similarity between histological images through graph-theoretic description and matching useful for the purpose of content-based retrieval. A higher order (region-based) graph-theoretic representation of histological images has been proposed and a tree-search based optimal matching algorithm has been employed. The proposed method facilitates the automatic retrieval of images structurally similar to a given image. Such a system can be used for several applications in the biological and medical field.

The method has been applied specifically for histological images. The reason behind the conception of the idea is the fact that the state-of-the art CBIR methods that differentiate images mostly in terms of low-level colour, shape and texture features do not perform well with histological images, as only these features are inadequate to capture the spatial content and neighbourhood relationships of histological images. The structural characteristics are very important to differentiate between morphological components in a particular tissue, and the method developed utilizes this fact to obtain similar tissue areas, of particular interest to the user.

It can be seen that the results obtained are satisfactory for histological images, as shown for the human breast in our study. The performance evaluation suggests that the technique developed is effective and superior to the simpler histogram-based technique. The execution time depends on the size and complexity of the query image selected by the user.

Future work on this system may include the incorporation of other appropriate attributes like Euler number, solidity etc. for nodes and the differences between properties like compactness for adjacent nodes as edge attributes in the graph-based representation of images. Additionally, the procedure for graph matching can be optimised from an application-oriented point of view so that the execution time for matching large sized graphs is further reduced.

Moreover, in the current study, the focus is on breast tissue biopsy images. The method can be generalised to other types of histological images or can be studied for new categories of images in which structure and spatial relationships are of major importance.

## Abbreviations

ARG: Attributed Relational Graph; CBIR: Content-Based Image Retrieval; CS: Common Subgraph; FEA: Flat Epithelial Atypia; GUI: Graphical User Interface; H&E: Hematoxylin and Eosin; IR: Information Retrieval; MCS: Maximum Common Subgraph; NP: Nondeterministic Polynomial time; PCA: Principal Component Analysis; RAG: Region Adjacency Graph; ROI: Region of Interest; WSI: Whole Slide Images.

## Competing interests

The authors declare that they have no competing interests.

## Authors’ contributions

Alexander Alekseychuk, Olaf Hellwich and Harshita Sharma participated in conception of the idea and design and coordination of the method. Harshita Sharma implemented the retrieval method, performed evaluation and drafted the manuscript. Alexander Alekseychuk developed and implemented the segmentation algorithm for preprocessing step and helped in drafting the manuscript. Peter Leskovsky and R. S. Anand also helped in drafting the manuscript. Peter Hufnagl and Norman Zerbe introduced medical background and provided whole slide images of breast biopsies together with annotated and classified learning samples for segmentation of tissue. All authors have read and approved the final manuscript.
